# Obstetric and perinatal outcomes for twin pregnancies in adolescent girls

**DOI:** 10.1038/s41598-018-37364-2

**Published:** 2018-12-24

**Authors:** Danielle Robson, Samuel Daniels, Christopher Flatley, Sailesh Kumar

**Affiliations:** 1 School of Medicine, The University of Queensland, Brisbane, Australia; 2 Mater Research Institute - University of Queensland, Level 3 Aubigny Place, Raymond Terrace, South Brisbane, Queensland, QLD 4101 Australia

## Abstract

This was a nine-year retrospective cohort study to investigate obstetric and perinatal outcomes in a cohort of adolescent girls with twin pregnancies from a major Australian tertiary centre in Brisbane, Australia. The adolescent cohort was aged <19 years and the control group was aged 20–24 years. The total study cohort comprised of 183 women. Of these, the adolescent cohort contained 29 girls (15.8%) and the control group comprised of 154 women (84.2%). Adolescent girls were less likely to delivery via an elective caesarean section compared to women in the control group (10.3% vs. 25.7%, p < 0.001). There were no differences in duration of labour, post-partum haemorrhage or perineal trauma rates. After controlling for the confounding effects of parity, chronicity and birth weight, birth <28 weeks remained significant (aOR 11.20, 95% CI 2.97–42.18, p < 0.001) for the adolescent cohort. There was a higher proportion of adolescents whose babies had an adverse composite perinatal outcome (87.9% vs. 69.5%, OR 3.20 95% CI: 1.40–7.31, p = 0.01) however significance was lost after adjusting for parity, chorionicity, birthweight and gestation at birth (aOR 3.27 95% CI: 0.95–11.31, p = 0.06). Our results show that obstetric and perinatal outcomes for twin pregnancies in teenagers were broadly similar compared to controls although the risk of extreme preterm birth was increased after controlling for confounders.

## Introduction

Adolescent pregnancy is defined as a pregnancy in girls aged 10–19 years and accounts for approximately 11% of births worldwide^1^. They are predisposed to obstetric complications such as obstructed labour, genital tract fistulae, postpartum haemorrhage, pre-eclampsia and anaemia^1–3^ more commonly observed in developing countries where rates of adolescent pregnancy are far higher.

Although the available literature on adolescent pregnancy in general is somewhat limited, there is a particular paucity of data of outcomes relating to multiple pregnancy. Regardless of maternal age, twin pregnancy is a risk factor for low birthweight and preterm birth^4^ as well as a myriad of other maternal complications. The aim of this study was to investigate obstetric and perinatal outcomes in a cohort of twin pregnancies in adolescent girls from an Australian tertiary centre.

## Methods

This was a nine-year cohort study of adolescents who birthed at the Mater Mother’s Hospital (MMH) in Brisbane, Australia between 1^st^ January 2007 and 31^st^ December 2015. The adolescent cohort was defined as any female aged 10–19 years and the control group consisted of women aged 20–24 years. Inclusion criteria were non-anomalous twin pregnancies >20 weeks gestation. Ethical, governance and waiver of consent approvals were granted by the Mater Human Research Ethics Committee and Governance and Privacy office (Reference: HREC/14/MHS/37 and RG-14–162) respectively and all methods were performed in accordance with relevant guidelines and regulations.

Socio-economic status was examined via the Socio-Economic Index for Areas (SEIFA) score. In Australia, a SEIFA score in the lowest quartile represents the most disadvantaged demographic group. Obstetric outcomes included onset of labour, mode and indication for birth, length of labour, blood loss and perineal trauma. Perinatal outcomes were gestation at birth, preterm birth, birthweight, birthweight <10^th^ centile (calculated for gestation and sex against Australian birthweight centiles). Serious composite perinatal outcome was defined as severe acidosis at birth (defined as cord pH < 7.1 or lactate >6 mmol/L or base excess <−12mmol/L) or death or admission to the NICU or Apgar score <7 at 5 minutes.

Univariate and multivariable analysis was performed using Generalised Estimating Equations to account for correlation between twins and to calculate odds ratios with 95% Confidence Intervals. Statistical analysis was performed using the Stata statistics program (StataCorp. 2015. *Stata Statistical Software: Release 14*. College Station, TX: StataCorp LP). Statistical significance was defined as p < 0.05.

## Results

Over the study period the total study cohort comprised of 183 women. The adolescent cohort comprised 29 girls (15.8%) and 154 women (84.2%) in the control group. There were 17 (58.6%) dichorionic diamniotic (DCDA) and 12 (41.4%) monochorionic diamniotic (MCDA) twins in the adolescent group and 65 (42.2%) DCDA and 89 (57.8%) MCDA twins in the control group respectively. Compared to the control group, adolescents were more likely to be nulliparous and not be married (Table 1).

**Table 1:** Maternal Demographics.

	*<20y*.*o (n* = *29)*	*20–24y*.*o (n* = *154)*	*P value*
Maternal Age	19 (18–19)	23 (22–24)	<0.001
BMI (kg/m^2^)			
<18.0	14/24 (58.3%)	66/141 (46.8%)	0.3
18.1–24.9	3/24 (12.5%)	9/141 (6.4%)	0.29
25.0–30.0	4/24 (16.7%)	30/141(21.3%)	0.61
>30.0	3/24 (12.5%)	36/141 (25.5%)	0.17
Model of care			
Public	27/29 (93.1%)	126/154 (81.8%)	0.18
Private	2/29 (6.9%)	28/154 (18.2%)	
Nulliparous	25/29 (86.2%)	100/154 (64.9%)	0.03
Assisted Reproduction	0	13/154 (8.4%)	NA
Ethnicity			
Caucasian	22/29 (75.9%)	114/154 (74.0%)	0.83
Indigenous	2/29 (6.9%)	7/154 (4.6%)	0.6
Other	5/29 (17.2%)	33/154 (21.4%)	0.61
Refugee	1/29 (3.5%)	2/154 (1.3%)	0.41
Marital Status			
Married/Defacto	10/24 (41.7%)	101/144 (70.1%)	
Not Married	14/24 (58.3%)	43/144 (29.9%)	0.01
Education			
Tertiary	0/25 (0%)	30/136 (22.1%)	0.01
Grade 10–12	22/25 (88.0%)	100/136 (73.5%)	0.12
<Grade 10	2/25 (8.0%)	6/136 (4.4%)	0.45
Smoking	11/29 (37.9%)	48/154 (31.2%)	0.52
Alcohol	4/29 (13.8%)	27/154 (17.5%)	0.79
Diabetes	1/29 (3.5%)	13/154 (8.4%)	0.7
Hypertension	3/29 (10.3%)	12/154 (7.8%)	0.71
Asthma	10/29 (15.9%)	29/154 (18.8%)	0.08
EPDS ≥ 12	2/18 (11.1%)	17/91 (18.7%)	0.73
SEIFA Score	1,017 (964–1,053)	1,006 (958-1,060)	0.97

EPDS – Edinburgh post-natal depression score, BMI – Body mass index.

SEIFA – Socio-economic index for areas.

After adjusting for parity, chorionicity, birth weight and gestation at birth intrapartum outcomes between the two groups were very similar. (Table 2) Although there were high overall rates of emergency caesarean section in both cohorts with almost one in two adolescent girls requiring this intervention this difference was not significant (48.3% vs. 38.6%, p = 0.17). Adolescent girls were less likely to delivery via an elective caesarean section compared to women in the control group (10.3% vs. 25.7%, p < 0.001). There were no differences in duration of labour, post-partum haemorrhage or perineal trauma rates.

**Table 2:** Maternal Outcomes.

	Adolescent Pregnancy (<20 y.o)	C Control Group (20–24 y.o)	p-value	Unadjusted Odds Ratio (95% CI)	p-value	Adjusted Odds Ratio* (95% CI)	p-value
Onset of Labour							
Spontaneous	11/17 (64.7%)	68/98 (69.4%)	0.78	0.92 (0.35–2.41)	0.86	1.23 (0.50–2.98)	0.65
Induced	6/17 (35.3%)	30/98 (30.6%)					
Method of Birth							
SVD	9/29 (31.0%)	45/154 (29.2%)	0.78	1.09 (0.59–2.00)	0.78	0. 94 (0.49–1.77)	0.84
Instrumental	3/29 (10.3%)	10/154 (6.5%)	0.3	1.66 (0.63–4.33)	0.3	2.01 (0.71–5.69)	0.19
Caesarean Section	17/29 (58.6%)	99/154 (64.3%)	0.41	0.79 (0.44–1.39)	0.41	0.85 (0.47–1.55)	0.61
Elective CS	3/29 (10.3%)	40/154 (25.7%)	<0.001	0.33 (0.14–0.81)	0.02	0. 47 (0.19–1.19)	0.11
Emergency CS	14/29 (48.3%)	60/154(38.6%)	0.17	1.48 (0.84–2.60)	0.17	1. 20 (0.66–2.18)	0.56
NRFS	1/29 (3.5%)	9/154 (5.8%)	0.48	0.58 (0.13–2.55)	0.47	0.49 (0.11–2.32)	0.38
FTP^§^	1/29 (3.5%)	5/154 (2.9%)	0.81	1.19 (0.25–5.64)	0.83	1.72 (0.32–9.22)	0.53
Other	12/29(41.4%)	46/154 (29.9%)	0.08	1.66 (0.93–2.95)	0.09	1.27 (0.68–2.38)	0.45
Total Length of Labour (min)	349 (188–430)	366 (201–565)	0.27	−107.1 (−511.0–296.8)	0.6	−164.3 (−595.3–266.7)	0.46
PPH >1000 mls	1/29 (3.5%)	7/153 (4.6%)	1	0.99 (0.11–8.74)	0.99	0.99 (0.11–8.74)	0.99
Blood Loss	500 (300–600)	500 (350–500)	0.51	−35.7 (−136.8–65.5)	0.49	−41.7 (−156.5–73.1)	0.48

*Adjusted for parity, chorionicity, Birth Weight and gestation.

Spontaneous Vaginal Delivery (SVD), Caesarean Section (CS), Non-reassuring Fetal Status (NRFS), Failure to Progress (FTP), Postpartum Haemorrhage (PPH).

Although adolescent girls had higher rates of preterm birth <32 weeks (48.3% vs. 23.7%, OR 3.00, 95% CI: 1.69–5.36, p < 0.001) and <28 weeks (27.6% vs. 6.2%, OR 5.79, 95% CI: 2.77–12.14, p < 0.001), after controlling for the confounding effects of parity, chronicity and birth weight, only birth <28 weeks remained significant (aOR 11.20, 95% CI 2.97–42.18, p < 0.001). The median gestation at birth and median birth weights were lower in the adolescent cohort compared to controls. However, teenage girls were less likely to deliver a twin infant with birth weight <10^th^ centile. There was a higher proportion of adolescents whose babies had an adverse composite perinatal outcome (87.9% vs. 69.5%, OR 3.20 95% CI: 1.40–7.31, p = 0.01), however significance was lost after adjusting for parity, chorionicity, birthweight and gestation at birth (aOR 3.27 95% CI: 0.95–11.31, p = 0.06). (Table 3) As only 3 adolescents were multiparous we also analysed the data for only the primiparous cohort (Tables 4–6) with essentially similar results.

**Table 3:** Neonatal Outcomes.

	Adolescent Pregnancy (<20y.o)	Control Group	Unadjusted Odds Ratio (95% CI)	p-value	Adjusted Odds Ratio (95% CI)	p-value
Monochorionic Twins*	12/29 (41.4%)	89/154 (57.8%)				
Dichorionic Twins*	17/29 (58.6%)	65/154 (42.2%)	1.94 (0.87–4.34)	0.11	1.67 (0.72–3.74)	0.23
Gestational Age at Birth	32.5 (27–35)	34 (32–36)		0.002		
Preterm <37 weeks^#^	23/29 (79.3%)	116/154 (75.3%)	1.26 (0.63–2.49)	0.52	0.79 (0.31–2.03)	0.62
Preterm <32 weeks^#^	14/29 (48.3%)	37/154 (24.0%)	3.00 (1.69–5.36)	<0.001	2.39 (0.91–6.23)	0.08
Preterm <28 weeks^#^	8/29 (27.6%)	10/154 (6.4%)	5.79 (2.77–12.14)	<0.001	11.20 (2.97–42.18)	<0.001
BWT (g)^¥^	1690 (970–2450)	2180 (1540–2562)	−306.2–512.7–−99.8)	0.004	77.40 (−44.14–198.95)	0.21
Twin 1^¥^	1680 (970–2290)	2259 (1680–2585)	−372.2 (−651.6–−92.8)	0.01	23.39 (−113.90–160.68)	0.74
Twin 2^¥^	1668 (1110–2480)	2091.5 (1435–2495)	−240.3 (−541.5–60.9)	0.12	130.76 (−62.72–324.23)	0.19
BWT <10th centile^£^	7/58 (12.1%)	74/308 (24.0%)	0.43 (0.19–0.997)	0.049	0.41 (0.17–0.95)	0.04
Apgar @ 5 mins <7	11/56 (19.6%)	18/294 (6.1%)	3.75 (1.66–8.46)	0.001	1.19 (0.50–2.87)	0.69
Acidosis	2/58 (3.5%)	11/308 (3.6%)	0.96 (0.21–4.47)	0.96	0.90 (0.18–4.56)	0.9
Respiratory Distress	21/58 (36.2%)	103/308 (33.4%)	1.13 (0.63–2.03)	0.68	1.11 (0.56–2.20)	0.77
NICU Admission	46/58 (79.3%)	192/308 (62.3%)	2.32 (1.18–4.55)	0.02	0.90 (0.38–2.11)	0.8
Died	7/58 (12.1%)	20/308 (6.5%)	1.98 (0.79–4.91)	0.14	1.38 (0.41–4.70)	0.6
Adverse Composite Perinatal Outcome*	51/58 (87.9%)	214/308 (69.5%)	3.20 (1.40–7.31)	0.01	3.27 (0.95–11.31)	0.06

*Adjusted for parity, chorionicity, Birth Weight, gestation at birth.

^#^Adjusted for parity, chorionicity, Birth Weight.

^¥^Coefficient reported - Adjusted for parity, chrionicity, gestation.

^£^Adjusted for parity, chorionicity.

Neonatal Intensive Care Unit (NICU), Birth Weight (BWT).

**Table 4:** Maternal Demographics (Primiparous cohort).

	<20 y.o (n = 26)	20-24y.o (n = 102)	P value
Maternal Age^¥^	19 (18–19)	23 (22–24)	<0.001
BMI	22.7 (20.2–25.3)	24.9 (21.3–30.1)	0.09
<18.0^£^	3/21 (14.3%)	9/91 (9.9%)	0.69
18.1–24.9^§^	11/21 (52.4%)	37/91 (40.7%)	0.33
25.0–30.0^£^	4/21 (19.1%)	19/91 (20.9%)	1.00
>30.0^£^	3/21 (14.3%)	26/91 (28.6%)	0.27
Model of care			
Public^£^	24/26 (92.3%)	72/102 (70.6%)	0.02
Private	2/26 (7.7%)	30/102 (29.4%)	
Assisted Reproduction^£^	0/26 (0%)	9/102 (8.4%)	0.20
Ethnicity			
Caucasian^§^	19/26 (73.1%)	76/102 (74.5%)	0.88
Aboriginal & Torres Strait Islander^£^	2/26 (7.7%)	5/102 (4.9%)	0.63
Other^£^	5/26 (19.2%)	21/102 (20.6%)	1.00
Refugee^£^	1/26 (3.9%)	2/102 (2.0%)	0.50
Marital Status^§^			
Married/Defacto	9/22 (40.9%)	68/94 (72.3%)	
Not Married	13/22 (59.1%)	26/94 (27.7%)	0.01
Education			
Tertiary^£^	1/22 (4.6%)	23/87 (26.4%)	0.04
Grade 10-12^£^	20/22 (90.9%)	62/87 (71.3%)	0.09
<Grade 10^£^	1/22 (4.6%)	2/87 (2.3%)	0.50
Smoking^§^	10/26 (38.5%)	25/102 (24.5%)	0.15
Alcohol^£^	0/26 (0%)	8/102 (7.8%)	0.36
Diabetes^£^	1/26 (3.9%)	8/102 (7.8%)	0.69
Hypertension^£^	3/26 (11.5%)	10/102 (9.8%)	0.73
Asthma^£^	10/26 (38.5%)	14/102 (13.7%)	0.01
EPDS ≥ 12^£^	2/16 (12.5%)	14/54 (25.9%)	0.33
SEIFA Index^¥^	1,015 (964-1,053)	1,012 (962-1,056)	0.68

Where the denominators do not match the total numbers in each cohort the difference indicate missing data or non-recorded data. ^£^Fishers Exact, ^§^Z- Test for two proportions, ^¥^Wilcoxon Rank Sum test, BMI – Body mass index, EPDS – Edinburgh post-natal depression score, SEIFA – Socio-economic index for areas.

**Table 5:** Maternal Outcomes (Primiparous cohort).

	Adolescent Pregnancy (<20y.o)	C Control Group	Unadjusted Odds Ratio (95% C.I.)	p-value	Adjusted Odds Ratio (95% C.I.)	p-value
		(20–24y.o)				
Onset of Labour*						
Spontaneous	20/26 (76.9%)	79/102 (77.5%)	1.03 (0.37–2.87)	0.95	1.45 (0.56–3.73)	0.45
Induced	6/26 (23.1%)	23/102 (22.6%)				
Method of Birth						
SVD*	18/52 (34.6%)	56/204 (27.5%)	1.40 (0.58–3.38)	0.46	1.23 (0.60–2.54)	0.57
Instrumental^##^	6/52 (11.5%)	19/204 (9.3%)	1.27 (0.37–4.35)	0.70	1.27 (0.37–4.35)	0.70
Caesarean Section*	24/52 (53.9%)	129/204 (63.2%)	0.68 (0.29–1.60)	0.38	0.65 (0.32–1.31)	0.23
Elective CS*	4/52 (7.7%)	51/204 (25.0%)	0.25 (0.06–1.12)	0.07	0.19 (0.04–0.86)	0.03
Emergency CS*	24/52 (46.2%)	78/204 (38.2%)	1.38 (0.59–3.25)	0.46	1.19 (0.59–2.40)	0.64
NRFS^##^	2/52 (3.9%)	9/204 (4.4%)	0.87 (0.10–7.51)	0.90	0.87 (0.10–7.51)	0.90
FTP^##^	2/52 (3.9%)	7/204 (3.4%)	1.13 (0.13–10.13)	0.92	1.13 (0.13–10.13)	0.92
Other*	20/52 (38.5%)	62/204 (30.4%)	1.43 (0.59–3.45)	0.42	1.05 (0.49–2.25)	0.90
Total Length of Labour (min)^¥^**	349 (188–430)	431 (208–605)	−164.6 (−677.4–348.1)	0.53	−158.7 (−662.6–345.3)	0.54
PPH >1000 mls^##^	1/26 (3.9%)	4/102 (3.9%)	0.98 (0.10–9.16)	0.99	0.98 (0.10–9.16)	0.99
Blood Loss^¥^**	500 (400–600)	500 (400–600)	−37.4 (−162.4–87.6)	0.56	−33.8 (−171.5–104.0)	0.63
Perineal Trauma^#^	6/26 (23.1%)	26/102 (25.5%)	0.88 (0.32–2.42)	0.80	0.39 (0.12–1.25)	0.11

C.I.: Confidence Interval; SVD: Spontaneous Vaginal Delivery; CS: Caesarean Section; NRFS: Non-reassuring Fetal Status; FTP: Failure to Progress; PPH: Postpartum Haemorrhage.

^¥^Coefficients Reported.

*Adjusted for Maternal Body Mass Index, Chorionicity, Birth Weight & Gestation.

**Adjusted for Maternal Body Mass Index, Chorionicity, Birth Weight, Gestation & Method of Birth.

^#^Adjusted for Birth Weight & Method of Birth.

^##^Unadjusted Odds Ratio’s Reported.

**Table 6:** Neonatal Outcomes (Primiparous cohort).

	Adolescent Pregnancy (<20y.o)	C Control Group	Unadjusted Odds Ratio (95% C.I.)	p-value	Adjusted Odds Ratio (95% C.I.)	p-value
		*(20–24y*.*o)*				
Monochorionic Twins	11/26 (42.3%)	57/102 (55.9%)	1.73 (0.72–4.13)	0.22	1.73 (0.72–4.13)	0.22
Dichorionic Twins^##^	15/26 (57.7%)	45/102 (44.1%)				
Gestational Age at Birth^**#**^	32.5 (27–35)	34 (31–36)	−1.61 (−2.80–0.41)	0.01	−0.97 (−1.68–0.27)	0.01
Preterm <37 weeks^**#**^	42/52 (80.8%)	156/204 (76.5%)	1.29 (0.60–2.77)	0.51	1.13 (0.39–3.28)	0.83
Preterm <32 weeks^**#**^	24/52 (46.2%)	62/204 (30.4%)	1.96 (1.05–3.65)	0.031	1.94 (0.63–6.01)	0.25
Preterm <28 weeks^**#**^	14/52 (26.9%)	14/204 (6.9%)	5.00 (2.21–11.34)	<0.0	24.14 (3.89–149.98)	0.001
BWT (g)^¥^	1,684 (1,040–2,415)	2,055 (1,423–2,514)	−233.1 (−527.7–61.5)	0.12	91.1 (−53.5–235.7)	0.22
Twin 1^¥^	1,735 (970–2,290)	2,173 (1,509–2,540)	−280.2 (−584.6–24.2)	0.07	10.3 (−145.8–166.5)	0.9
Twin 2^¥^	1,684 (1,110–2,480)	1,967 (1,400–2,428)	−186.0 (−502.2–130.2)	0.25	171.8 (−30.9–374.6)	0.1
BWT <10%^£^	7/52 (13.5%)	47/204 (23.0%)	0.52 (0.21–1.28)	0.16	0.36 (0.12–1.04)	0.06
Apgar @ 5 mins <7*	12/52 (23.1%)	23/204 (11.3%)	2.36 (0.93–6.02)	0.07	1.50 (0.56–4.01)	0.42
Acidosis^##^	2/52 (3.9%)	7/204 (3.4%)	1.13 (0.20–6.51)	0.9	1.13 (0.20–6.51)	0.9
Respiratory Distress**	28/52 (53.9%)	99/204 (48.5%)	1.24 (0.59–2.58)	0.57	1.01 (0.47–2.15)	0.98
NICU Admission**	42/52 (80.8%)	140/204 (68.6%)	1.92 (0.69–5.31)	0.21	1.18 (0.47–2.96)	0.73
Died^¥¥^	6/52 (11.5%)	15/204 (7.4%)	1.64 (0.46–5.91)	0.45	1.29 (0.31–5.28)	0.73
Adverse Composite Perinatal Outcome**	47/52 (90.4%)	156/204 (76.5%)	2.89 (0.75–11.17)	0.12	1.74 (0.25–12.41)	0.58

^#^Adjusted for BMI, Chorionicity & Birth Weight.

^¥^Coefficient reported - Adjusted for BMI, Chorionicity & Gestation at Birth.

^£^Adjusted for BMI & Chorionicity.

*Adjusted for Chorionicity, Birth Weight & Gestation at Birth.

**Adjusted for BMI, Chorionicity, Birth Weight, Gestation at Birth & Method of Birth.

^¥¥^Adjusted for Chorionicity, Birth Weight, Gestation at Birth & Method of Birth.

^##^Unadjusted Odds Ratio Reported.

C.I.: Confidence Interval; NICU: Neonatal Intensive Care Unit; BWT: Birth Weight

Acidosis defined as defined as cord pH < 7.1 or lactate >6 mmol/L or base excess <−12mmol/L.

Adverse Composite Perinatal Outcome: Acidosis, NICU admission, Apgar Score <7 at 5 minutes, Death.

## Discussion

The key finding of this study of multiple pregnancy outcomes in adolescents is the risk of extreme preterm birth (<28 weeks) after controlling for the potentially confounding effects of parity, chorionicity and birthweight. Although there was a higher proportion of neonates with the composite adverse outcome in the adolescent group this did not reach statistical significance after adjusting for confounders. We also found a decreased risk of low birth weight (birth weight < 10^th^ centile for gestation and gender) (aOR 0.41 95% CI: 0.17–0.95, p = 0.04). Although approximately 90% of the adolescent cohort was primiparous, there were no differences in outcomes when this cohort was separately analysed.

There is good evidence that the risk of perinatal death is substantially higher in adolescents compared to women aged 20 to 24 years of age^5^. One reason for this, is likely to be the higher rates of preterm birth as seen in our study. The causes for the higher rate of birth <28 weeks in the adolescent cohort are not immediately apparent. Furthermore, despite the higher rates of preterm birth in adolescent girls in this study appeared to be less likely to deliver a baby under the 10^th^ centile after controlling for parity and gestation at birth. This is in contrast to outcomes in teenage girls with singletons where there is a higher rate of low birth weight babies^6^.

Although we demonstrate overall comparable neonatal outcomes for adolescents with twin pregnancies albeit with some specific differences, earlier studies have suggested that rates of adverse outcomes in teenagers are no higher compared to controls^7^. Although there is evidence showing that overall perinatal outcomes for singletons are poorer in teenagers^8^ our results suggest that when confounders such as parity, chorionicity and gestation at birth are taken into account, outcomes are also poor in adolescents with multiple pregnancy. It is possible that these poorer pregnancy outcomes may be attributable to the sub-optimal pre-pregnancy health status of teenage girls as well as factors consistent with higher prevalence of poor socio-economic status in this cohort.

We did not observe poorer obstetric or intrapartum outcomes in our study. Overall caesarean section rates were high in both cohorts and there were no differences in total length of labour, rates of perineal trauma or postpartum haemorrhage. This is consistent with other published data^7^. Adolescents generally have higher rates of pregnancy complications including hypertensive disorders, antepartum haemorrhage, cephalopelvic disproportion and intervention for obstructed labour^1,2^. Consistent with other studies^9^ we found lower rates of elective caesarean section in the adolescent cohort suggesting that they were more amenable to attempting a vaginal twin delivery. This is important as there is evidence that adolescent younger mothers are far more likely to have further children in adolescence and thus more children overall in their lifetime^10^. As adolescents are more likely to develop post-natal mental health issues^11^ and difficulties with breastfeeding^12^ which are known to be associated with caesarean birth, any reduction in operative rates could potentially mitigate these important post-partum issues.

While rates of adolescent pregnancy have declined^13^, complications of pregnancy and childbirth are the second leading cause of death for girls aged 15–19 years old^13^. As such, the importance of education and universal access to contraception is an important priority in addressing overall disparities in obstetric and perinatal outcomes regardless of the number of fetuses. Indeed, there is evidence to suggest that the risks for adverse pregnancy outcomes in teenage pregnancies can be mitigated by high-quality maternity care^14^.

The strengths of our study are the inclusion of clinically relevant outcomes and the adjusting for relevant confounders such as chorionicity, parity, gestation at birth and birthweight. The limitations were related to the relatively small number of cases of only 29 teenagers, however the literature is extremely limited with only one paper from 1990^7^ addressing this subject. Although overall, pregnancies in adolescent are not uncommon, multiple pregnancy is and there is a lack of information regarding pregnancy outcomes. We were unable to ascertain termination of pregnancy and miscarriage rates, outcomes that are pertinent to the study cohort. Our results are clinically relevant, highlighting the importance of engaging adolescents and supporting them both during and outside of pregnancy.
